# Determinants and the impact of the National Health Insurance on neonatal mortality in Ghana

**DOI:** 10.1186/s13561-017-0169-z

**Published:** 2017-09-29

**Authors:** Monica Lambon-Quayefio, Nkechi S. Owoo

**Affiliations:** Economics Department, University of Ghana, P.O.BOX LG 57, Accra, Ghana

**Keywords:** Health insurance, Neonatal deaths, Health care access, Ghana

## Abstract

The national health insurance was established to increase access to health care services and the maternal component was later introduced to improve the health outcomes of both mother and child. The main objectives of this study are to investigate the factors that affect neonatal deaths as well as examine the effect of the Ghana Health Insurance on neonatal deaths in Ghana. Using the most recent round of the Ghana Demographic and Health Survey, the study estimates the probit model with interaction effects to account for the heterogeneity in outcomes. Additionally, the study employs the propensity score matching approach to account for the possible endogeneity in the insurance enrolment decision. Results from the estimations, after controlling for relevant individual and household characteristics suggest that the national health insurance significantly reduces the likelihood of neonatal deaths. Estimates remain consistent even after more robust estimators are employed. Estimates from the interaction between place of residence and health insurance indicate that health insurance beneficiaries who reside in urban areas are at a higher risk of neonatal deaths compared to other women. Access to medical facilities proxied by distance to the nearest health post emerged as an important predictor of neonatal death. The study also suggests significant regional differences in neonatal deaths. We, therefore, conclude that the national health insurance may have the potential to substantially improve the health outcomes of neonates and have policy implications for increasing coverage to more mothers and their neonates, as well as coverage in critical neonatal services and drugs.

## Background

The neonatal period- the first 28 days- is the most vulnerable period for the survival of every child. A new-born dies every 15 minutes in Ghana according to recent data from United Nations Children Emergency Fund (UNICEF, 2015). This reflects the relatively high levels of neonatal mortality recorded in the country. Globally, despite the accelerating progress made towards child survival, the decline in neonatal mortality has been slowest from the period 1990 to 2015. As such, the proportion of newborn deaths in child mortality has increased from about 37% in 1990 to 44% in 2013 (United Nations Interagency Group Child Mortality Estimation-UN-IGME, 2015).

After declining steadily from 122 deaths per 1000 live births in 1990 to 98 deaths per 1000 live births in 1998, the under-five mortality appeared to have stagnated at 111 deaths per 1000 live births between 2003 and 2008 [1]. The main reason for this reversal is the increased neonatal mortality (Ghana Newborn Health Strategy and Action Plan, 2014). Like the rest of the world, Ghana has experienced a stagnation in the declines in neonatal deaths. Data from the Ghana Health Service (GHS, 2010) show some inconsistency in the decline in neonatal mortality from 1993 through to 2015. The neonatal mortality rate was 41 deaths per 1000 live births in1993, declining to 30 deaths per 1000 live births, rising to 43 in 2003 and then falling again to 30 deaths per 1000 live births in 2008. UNICEF (2015) reports the 2013 neonatal mortality rate to be 29.3 and the 2015 rate to be 32 deaths per 1000 live births. The sluggish rate of neonatal declines since 1998 has resulted in the increase in neonatal deaths’ contribution to infant deaths from 53% in 1998 to about 71% in 2014 (Ghana Demographic and Health Survey, 2014) as well as its contribution to child mortality. Currently, neonatal mortality contributes about 40% of child mortality. As a result, neonatal mortality has become a very important component of infant mortality and child mortality and requires very exigent attention (Ghana National Newborn Health Strategy, 2014).Generally, aside other biological and medical factors, lack of access to modern health care services and medication are major contributing factors to child mortality [2]. High user fees and out-of-pocket payments which characterize most developing countries have generally been associated with low health care utilization. In developing countries like Ghana lack of access to health services due to financial constraints and/or physical access may result in delays in seeking treatment and encourage self-treatment, which are both associated with increased health complications [3]. In most cases, it hampers access to modern health services especially for young children [4].

In Ghana, the Newborn Health Strategy and Action plan whose goal is to reduce neonatal mortality from 30 to 21 deaths per 1000 live births by 2018 identified the issue of health financing for newborn care as one of the challenges which hinders improvement in the health of newborns. Although the national health insurance is currently designed to cover all pregnant women and children under 18 years (which includes newborns), not all services are covered by the scheme. This study, therefore, aims to investigate the risk factors of neonatal deaths and examine the potential role the national health insurance scheme can play in reducing the rate of neonatal mortality in Ghana. Since its establishment, only two studies have directly examined the effects of the health insurance on neonatal health. Both studies [5, 6] find a positive relationship between the national health insurance and new born health. Yet, these studies are very limited in terms of geographic coverage and measurement of new born health. The first study focused on only two districts out of the 216 districts in Ghana and employs post-natal checks as a proxy for new born health. Although the second study makes use of a nationally representative data, it also uses the continuum of maternal care as a proxy measure of new born health and does not account for endogeneity in the health insurance enrolment decision. To the best of our knowledge, no other study has examined the effect of the national health insurance scheme by simultaneously using more direct measures of neonatal mortality, using nationally representative data and accounting for endogeneity. This study, therefore, adds to the limited knowledge stock in the area of neonatal research.

This study may also have important implications for newborn health policy in Ghana. The Newborn Health Strategy and Action plan outlined strategies to substantially reduce out-of-pocket payments for essential drugs and tests for newborns as a way of reducing the financial barriers to accessing newborn health services. Another strategy is to increase advocacy efforts to improve the national health insurance’s coverage on neonatal related health care. As such, findings from this research will provide the required empirical evidence to inform policy changes on increasing coverage to essential neonatal health needs. The remainder of the paper is organized in six sections as follows: the next section provides a brief historical background to the national health insurance, followed by section three which reviews existing literature on health insurance and child mortality. Section four describes the data while section five discusses the methodology used in the study. Section six provides the results and discussions while section seven concludes the paper with some policy recommendations.

### Historical background to the National Health Insurance in Ghana

National health insurance in Ghana dates back to post-independence. Healthcare during the period was entirely free and was wholly financed by government tax revenue [7]. It insulated the poor and marginalized from financial distress. However, in 1970, free health care provision was no longer sustainable as a result of inadequacy of resources and budgetary constraints [8]. Consequently, a statutory dispensing fee of 30np (New Pesewa) was introduced by the National Liberation Council (NLC [7]. Also, upon the enactment of the Hospital Fees Decree 1969 which was later amended into the Hospital Fees Act 1971, a minimal user fee was charged to cover hospital procedures.

The economic decline coupled with inflationary pressures and growing unemployment in Ghana, at the time, made it heed to the then seemingly attractive proposals implementing a structural adjustment program in 1983. The goal of this program was to withdraw all forms of subsidies and then liberalize the economy. Consequently, the full cost recovery (also known as cash-and-carry) was introduced and adopted into the health system with the introduction of the Hospital Fees Regulation 1985 (L.I.1313). This extended the fees charged to include consultation, laboratory and other diagnostic procedures, medical, surgical and dental services, medical examinations and hospital accommodation [9]. Post-recovery programme’s government expenditure on the health sector dropped to less than 20% that of the pre-1970.

Recognizing the importance of health care to the quality of human capital, Ghana made several attempts at finding alternatives that will abolish the cash and carry system. Various experimented alternatives proved unsuccessful, largely because of lack of resources and the inability of the government to pay for the budget of the health sector. Finally, the government took the decision to experiment with a social health financing initiative which led to the introduction of a health insurance scheme on a pilot basis in the 1990s. Afterwards, community health insurance (CHI) schemes and Mutual Health Organizations (MHO) sprang up in Ghana. They were mainly funded by faith-entities and international organizations [9].

### The National Health Insurance

The development of the current National Health Insurance Scheme commenced with Ghana’s resolve to access the highly indebted poor country (HIPC) initiative in March 2001. The government of Ghana was to allocate the funds towards projects that sought to reduce poverty and enhance economic growth. Simply, it highlighted the protection of the poor and marginalized, with special reference to women and children. As a result, in February 2003, some amount of the HIPC fund was allocated by the Ministry of Health to support the running and creation of Mutual health Organizations in most parts of the country. The national health insurance bill (Act 650) was eventually placed before the legislature for considerations. The bill required the formal and the informal sector to enroll together in government-sponsored district MHOs [8, 9].

The national health insurance scheme is financed from a pool of sources of which the individual premium payments ranging from GH7.2 to 48.0 (roughly USD 3.6 to 24) per person per year. These premiums are progressive which means the rich pays more than the poor. This source finances less than 5% of the NHIS expenditure (NHIA,2013). According to NHIA [10], the major source of funding is the value-added tax of 2.5% on all goods and services, which contributes approximately 60% to the NHIS revenue. Other funding sources include investment income or interest earned on National Health Insurance Fund reserves (17% of NHIS revenues), a 2.5% of social security contributions from formal sector workers (16% of NHIS revenues).

Almost all outpatient and inpatient services targeting over 90% of the disease burden including essential medicines (as included in the NHIS approved list) are offered to the insured without any co-payments. The insurance is cashless and the insured are not required to make any payment at the time of health care delivery. Payments for referrals up to the teaching hospitals are covered. However, HIV retroviral drugs, hormone, and organ replacement therapy, heart and brain surgery other than the ones caused accidents, diagnosis, and treatment abroad, dialysis for chronic renal failure and cancers are excluded from the insurance package [11].

### Health service access and delivery under the NHIS

The National Health Insurance Scheme, which was implemented in 2004, has been accepted by Ghanaians as one of the best homegrown social intervention programs to be introduced in the country. Research reveals that the National Health Insurance Scheme has expanded access to health care for the majority of the population who, until its introduction, could not afford health care under the ‘cash and carry’ system. As at the end of December 2011, the total active membership of the scheme increased from 8.16 million in 2010 to 8.23 million in 2011 showing an increase of 0.8% over the 2010 figure and representing 33% of the population [9].

Moreover, upon the institution of the NHIS, outpatient utilization augmented by over twenty-eight fold from 0.6 million in 2005 to 16.9 and 25.5 million in the year 2010 and 2011 respectively. Similarly, Inpatient utilization increased over 30 fold from 28,906 in 2005 to 973,524 in 2009 but dropped to 724,440 in 2010. In 2011, however, inpatient utilization doubled, from 724,440 to 1.45 million. The Free Maternal Care (FMC) component was introduced in July 2008 to contribute to meeting the Millennium Development Goals (MDG) 4 and 5. Under this program, pregnant women are to receive free medical care. However, due to abuse of the system, the National Health Insurance Authority (NHIA) revised the implementation guidelines in 2010 to encourage pregnant women to register with the scheme before accessing health care [12]. Yet, it was revealed that the poor quality of the health care offered to NHIS card holders served as disincentive for pregnant women to register with the scheme [9].

### Neonatal, infant and child health care under the NHIS

Though the NHIS insured beneficiaries against various health risks, the benefits of maternal and neonatal care under the scheme were central. In the second half of 2008, the revised free maternal care program was implemented under the NHIS, which mandated pregnant women to be enrolled onto the scheme (without payment of premiums) in order to enjoy the program benefits. The program allowed all expectant mothers to enjoy free health care services right from conception till after delivery. They could similarly access postnatal care at accredited heath facilities at no cost. The benefit package also included free neonatal coverage. Specifically, neonates enjoy free health services on the mother’s card three months after delivery [13] Additionally, all babies had the right to free basic healthcare for a whole year [12, 14], and nationals below age 18 were also entitled to free health care under the scheme [15]. Consequently, a 30% reduction in infant mortality rate and another 13% decline in neonatal deaths were recorded between 2008 and 2011 [16, 17].

## Literature review

Improving women’s access to quality health care during pregnancy and infant and children’s access to essential health care services is imperative for improved maternal and child health. The relationship between out of pocket payments for health care and child health outcomes have been documented by [18, 19]. There is however very little evidence about the relationship between health insurance and neonatal mortality because very few studies have measured these outcomes [20]. Globally, evidence from a systematic review by Spaan et al. [21] suggest that health insurance increases health care utilization. In Ghana, a number of studies [6, 22, 23] have confirmed the positive relationship between the health insurance and health care utilization, since its implementation in 2005 in terms of improvement in maternal and child health outcomes. Using a nationally representative data, Owoo and Lambon-Quayefio [24] confirmed a positive relationship between health insurance and antenatal visits. Brugiavini and Pace [25] and Mensah et al. [6] have also confirmed that women who are enrolled on the health insurance scheme are likely to deliver in institutions compared to women who did have health insurance. Access to and utilization of health care have been described by Comfort et al. [20] to be mediating factors of the impact of health insurance on health outcomes.

With respect to its link with newborn health and child mortality, most of the available evidence is based on studies from relatively more advanced countries which provide inconclusive findings. Some of these studies [26–29] have employed natural and quasi-natural experiments while others such as [5] have also relied on non-experimental econometric techniques in evaluating the effect of health insurance on child survival.

The Medicaid program in the United States was found to significantly reduce infant mortality and low birth weights, according to [30, 31] who used the instrumental variable approach to account for the endogeneity in the health insurance uptake decision. The State Children’s Health Insurance program has also been linked with improved child health outcomes in the United States according to Joyce and Racine [32]. Similarly, in Taiwan, Chou et al. [33] relied on the difference in difference approach to evaluate the effect of health insurance on birth outcomes. Results showed a positive impact on birth outcomes and child mortality. However, these studies have focused more on post-neonatal mortality rather than on neonatal mortality. The limited evidence available on health insurance and neonatal mortality suggest a negative relationship. Using data for Brazil, Barros et al. [34] focused on gestational specific neonatal mortality and birthweight specific neonatal mortality and found that neonatal mortality decreased with coverage of health insurance.

Ghana specific studies on health insurance and neonatal health is even more limited. Mensah et al. [6] attempted to explore this relationship using the propensity score matching technique to account for the self-selection of women enrolling onto the national health scheme. The study finds a positive relationship between health insurance and newborn outcomes. The limitation of the study is two-fold. First, the study used post-natal care as a proxy for newborn health. However, whether or not mothers attend post-natal visits may not accurately capture the health status of neonates. Secondly, even though the study employed a rigorous estimation technique, the data employed focused only on two out of the 216 districts in Ghana. As a result, the findings from this study may not be nationally representative. Similarly, Browne and Kayode et al. [5] also used maternal continuum of care services such as antenatal care, skilled deliver and postnatal care as proxies for newborn health. However, Browne and Kayode et al. [5] do not account for the endogeneity in the health insurance enrolment decision. The objective of the study is two fold. The first is to examine the factors that influence neonatal deaths in Ghana, and the second is to estimate the impact of the Ghana’s national health insurance on neonatal deaths. The first objective aims to update the neonatal mortality literature in Ghana’s context, examining which factors significantly correlate with neonatal deaths, using more recent data. The second objective of the study adds to the limited evidence available on the association between health insurance and newborn health in two ways- using more direct measures of neonatal mortality and accounting for endogeneity, using a nationally representative data.

## Data

The study makes use of the 2014 Ghana demographic and health survey which is a nationally representative data constituting over 12,000 households. The sampling for this data is based on a two-stage sampling technique. In the first stage, a total of 427 clusters were selected covering both the urban and the rural areas. From these clusters, 30 households were systematically selected in each of these clusters. Data is collected using three main questionnaires namely, the household questionnaire, the men’s questionnaire and the women’s questionnaire. Specifically, the analysis makes use of information from the women’s questionnaire which captures demographic and socioeconomic information on women within their reproductive ages (15–49). The data includes information on birth history dating 5 years preceding the survey. The data also contains other relevant information such as details on education, wealth, employment, marital status as well as information on whether or not the household members have valid national health insurance. The main variable of interest in this analysis is whether or not the individual woman has a valid national health insurance card. Table 1 below provides a detailed description of the variables used in the study while Table 2 provides the summary statistics of the variables used in the analysis.

**Table 1:** Detailed description of study variables

Variable Name	Type	Constructed from:
Neonatal Death	Dummy (1-if age at death is 1 month;0 otherwise)	Age at death in months
*Health Insurance Status* No Valid NHIS (base) Valid NHIS	Dummy (1 if individual has a valid NHIS;0 otherwise)	Does individual hold a valid health insurance card?
*Age of Mother* Mother’s age	Continuous	Mother’s current age
*Birth type* Single (base) Twin	Dummy (1- twin birth; 0 single)	Child is twin
*Birth Order of Child* Birth Order	Count	Birth order of child
*Sex of Child* Child is Female(base) Child is Male	Dummy (1 male;0- female)	Sex of Child
*Antenatal Care Visits* Number of ANC visits	Continuous	Number of antenatal visits during pregnancy
*Delivery type* Natural birth (base) Caesarian birth	Dummy (1- delivery by c-section; 0 – natural delivery)	Delivered by caesarian section
*Birth Interval* Less than 2 yrs. (base) 2 yrs. or more	Dummy (1- > = 2 yrs.; 0- <2 yrs)	Preceding birth interval
*Distance to Health Facility* Not a big problem (base) A big problem	Dummy (1- distance a problem; 0 distance not a problem)	In getting medical help, is distance a big problem
*Place of Delivery* Non-Facility delivery (base) Facility delivery	Dummy (1- delivered in a health facility; 0 otherwise)	Place of delivery
Distance to Health Facility		
*Employment Status* Unemployed (base group) Employed	Dummy(1 employed;0 unemployed)	Respondent worked in the last 12 months
*Education:* No Education (base group) Primary Education Secondary Education High Education	Categorical	Highest educational level of individual
*Marital Status* Single (base) Married Living together Formerly Married (widowed/divorced/separated)	Categorical	Current marital status
*Wealth Quintiles* Poorest (base group) Poor Middle Rich Richest	Categorical	Wealth index of household
*Location* Rural (base) Urban	Dummy (1- urban; 0 rural)	Type of place of residence
*Region of Residence* Greater Accra Western Central Volta Eastern Ashanti Brong-Ahafo Northern Upper East Upper West	Categorical	Region of residence

**Table 2:** Summary statistics

Study variables	Full sample		Valid Nhis		No Valid Nhis		Mean diff
	Mean	SD	Mean	SD	Mean	SD	*P*-value
Neonatal Death	0.04	0.19	0.04	0.19	0.04	0.19	0.642
Mother’s Age	36.86	7.62	37.43	7.63	36.55	7.58	0.000
Twin	0.04	0.19	0.03	0.17	0.04	0.19	0.095
Birth order	2.95	1.93	3.09	2.02	2.83	1.86	0.000
Child is Male	0.51	0.5	0.53	0.5	0.52	0.5	0.208
Number of ANC Visits	6.26	2.82	5.97	2.73	6.54	2.67	0.00
Delivered by CS	0.1	0.31	0.1	0.3	0.11	0.32	0.349
Faciltiy Delivery	0.92	0.27	0.9	0.3	0.93	0.25	0.000
Birth Interval	0.86	0.34	0.87	0.34	0.87	0.33	0.465
Distance to Health Facility a Problem	0.33	0.47	0.38	0.48	0.3	0.46	0.000
*Mother’s Education*							
No education	0.42	0.49	0.57	0.5	0.4	0.49	0.000
Primary	0.2	0.4	0.16	0.37	0.19	0.39	0.001
Secondary	0.35	0.48	0.25	0.44	0.38	0.49	0.000
Higher	0.02	0.15	0.02	0.14	0.03	0.17	0.014
*Employment Status*							
Employed	0.87	0.33	0.9	0.29	0.86	0.34	0.000
*Marital Status*							
Single	0.03	0.17	0.02	0.15	0.03	0.17	0.079
Married	0.69	0.46	0.75	0.43	0.73	0.45	0.011
Living Together	0.15	0.36	0.11	0.31	0.14	0.34	0.001
Formerly Married	0.13	0.34	0.12	0.32	0.11	0.31	0.339
*Wealth Quintiles*							
Poorest	0.32	0.47	0.47	0.5	0.32	0.47	0.000
Poor	0.23	0.42	0.16	0.37	0.22	0.41	0.000
Middle	0.2	0.4	0.16	0.36	0.2	0.4	0.000
Rich	0.14	0.35	0.09	0.29	0.15	0.36	0.000
Richest	0.11	0.31	0.12	0.33	0.12	0.33	0.911
*Place of Residence*							
Urban	0.397	0.49	0.42	0.49	0.37	0.48	0.000
*Region of Residence*							
Greater Accra	0.07	0.26	0.14	0.35	0.05	0.23	0.000
Western	0.1	0.3	0.05	0.21	0.1	0.31	0.000
Central	0.1	0.3	0.07	0.25	0.07	0.25	0.822
Volta	0.08	0.28	0.02	0.13	0.1	0.3	0.000
Eastern	0.1	0.3	0.07	0.25	0.1	0.3	0.000
Ashanti	0.11	0.31	0.01	0.09	0.1	0.3	0.000
Brong Ahafo	0.11	0.31	0.01	0.09	0.14	0.34	0.000
Northern	0.14	0.35	0.37	0.48	0.12	0.32	0.000
Upper East	0.1	0.3	0.02	0.15	0.12	0.32	0.000
Upper West	0.09	0.29	0.25	0.43	0.1	0.31	0.000
*Observations*	23,118		1941		13,055		

From the sample neonatal deaths make up about 4%. Disaggregating by national health insurance holders, there seem to be no statistical difference in the proportions of neonatal deaths among individuals who had valid health insurance and those that did not. The average age in the study sample is about 37 years. The difference in age between those with health insurance and those without is statistically significant at 1%. Although statistically significant only at 10% the data shows a higher incidence of multiple births among individuals with no valid national health insurance. About half (51%) of the children considered in this sample are male. On the whole, the average number of antenatal sessions attended is 6. The data also suggests a significantly lower antenatal attendance for mothers with NHIS compared with mothers without. Generally, almost all the women (92%) in the study reported delivering at a health facility. Comparatively, there were less facility deliveries among individuals with the valid health insurance cards. Given its importance to delivery outcomes, the issue of whether distance to the nearest health facility is a problem was included in the analysis. On the whole about a third (33%) of the respondents in the sample complained that distance to the nearest health facility was a big problem. The problem seem to be more pronounced among respondents with valid health insurance. Of those with valid health insurance, about 38% of them expressed worry about distance to the nearest facility compared to the 30% who had no valid health insurance.

The education and household wealth of respondents are also important determinants of risk of death of neonates. The data categorizes individual women’s education into four distinct groups namely no education, primary education, secondary education and higher education. As shown in the table, for all education categories considered in this study there are statistically significant differences between women with valid health insurance and those without. Overall, about 42% of the sample have no education. However, more than half (57%) of respondents with NHIS have no education compared to only 40% of women with no NHIS. Overall, 20% of the women in the sample have primary education. The proportion of women with NHIS who have primary education is about 16% compared to the 19% recorded among women with no valid health insurance. In general, approximately 35% of women in the study have secondary education. For women with valid nhis cards, the percentage of women with secondary education is about 25% while proportion is significantly higher for women with valid NHIS at 38%. Overall, only a few (2%) of the women have higher education. Among NHIS beneficiaries and non-beneficiaries, the proportion is approximately 2% and 3% respectively. Majority of the women (89%) reported as being employed. The proportions are similar among the sub groups of women with valid health insurance and no valid health insurance.

Based on information collected on ownership of various items and assets the survey generates wealth scores from which households are classified. According to the sample, women who belong to households categorized as being poorest, poor, middle, rich or richest categories are respectively 32%, 23%, 20%,14% and 11%. Consistently across the five quintiles, there are significant differences between women with valid health insurance and those with no valid health insurance. For instance, 47% of women who have valid NHIS are found in the poorest wealth category while 32% of women with no valid NHIS are also found in the poorest category. Similarly, for all the other four wealth categories, there are significantly higher proportions among women with no valid health insurance compared with women with valid health insurance. The study also controls for household location. The study accounts for the whether or not the respondents reside in the urban or rural area as well as region of residence in order to account for region specific characteristics.

## Methods

The study employs two empirical techniques for each of the research objectives. The first objective of examining factors that influence neonatal deaths makes use of the probit model due to the binary nature of the dependent variable. The form of the probit model estimated is as follows:

$$ Neonatal=\alpha NHIS+\beta Socioeconomic+\gamma Child+\varepsilon $$

Where *Neonatal* is a binary variable equal to 1 if the child died in the neonate stage and zero otherwise. *NHIS* is the variable of interest which captures whether or not the individual (mother) has a valid health insurance card or not. *Socioeconomic* is a vector that captures mother’s demographic and socio economic characteristics such as age, education, wealth, marital status as well as region and area of residence. *Child* is a vector of child specific characteristics such as gender and birth order. The coefficient, *α* merely measures the probability of neonatal mortality if a mother has a valid health insurance and not necessarily the impact of the health insurance on neonatal death as this would provide biased measure due to the potential endogeneity in the health insurance uptake decision.

According to Berk [35] and Nichols [36], selection bias occurs when relevant covariates, whether observable or unobservable are omitted in the analysis. This omission creates a situation where the explanatory variables are correlated with the residuals, thereby producing biased estimates and consequently affecting the reliance of the probit estimates for causal inferences. For example, the probit estimates may be overstating the effects of health insurance on the probability of neonatal mortality even after controlling for other relevant covariates. In the NHIS enrolment decision and as argued by Wang et al., [37] in a related study, women who enroll in the NHIS may differ significantly from women who do not. For example, women who are at a higher risk of losing their neonates may be more likely to enroll on the health insurance scheme. Women’s enrolment on the scheme is therefore not random. For example, some unobserved characteristics of women (perhaps specific health conditions) that make then more risky to lose their neonates also makes them risk averse. These may motivate them to enroll on the scheme. The unobserved heterogeneity in the characteristics of women in the sample may lead to unobserved selection bias.

Also, evidence from health care utilization studies, such as Arthur [38] and Dixon et al. [39] have indicated the rural-urban dichotomy in the utilization of maternal health care. As a result of this dichotomy, this study attempts to test the heterogeneity in the risk of neonatal death, by taking an interaction between the NHIS and whether or not the individual lives in the rural or urban areas.

The endogeneity problem is addressed by employing the Propensity Score Matching technique which is discussed below.

**Fig. 1:**
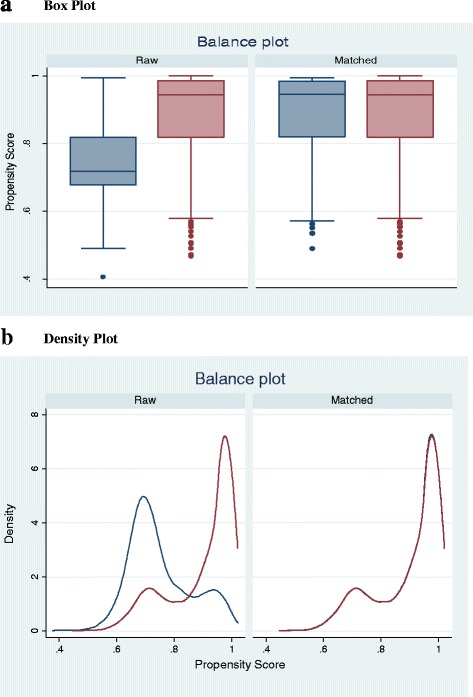
Balance plots of control and treated samples

The Propensity Score Matching technique developed by Rosenbaum and Rubin [40] has been described as an alternative to obtaining unbiased estimates in assessing program effects [41, 42]. In this technique propensity scores which are defined as the probability of assignment to the treated group, conditional on observed covariates are estimated. This balancing score is estimated based on a logit or probit regression where the treated and control subjects are then grouped based on similar propensity scores. The propensity scores then allows for the estimation of the average treatment on the treated [43]. This precisely allows for measurement the effect of the intervention or treatment. Despite the advantage of being able to directly estimate the treatment or program effect, the propensity score matching technique makes an assumption that unobservable differences does not exist between the treated and control groups [44] and as such does not balance on the unobserved characteristics. Table 3 below provides details of the balancing process for the study variables . The results of the overidentification test also suggest that both control and treatment groups are balanced. Also, the balance plots in Fig. 1 below indicate that the control and treatment groups are fairly similar.

**Table 3:** Propensity Score Balance Summary

A. Covariance Balance Summary				
	Raw		Matched	
Number of Observations	2905		5088	
Treated Observations	2544		2544	
Control Observations	361		2544	
Variable Name	Standardized Differences		Variance Ratio	
	Raw	Matched	Raw	Matched
Mother’s age	−.1296483	.0364344	.9287937	1.009999
Child is Twin	−.0208939	−.1156947	.8807093	.5463699
Birth Order	−.1119146	.0710256	.8888037	1.199043
Male	−.0085316	−.0559324	.9982579	1.006856
# ANC Visits	.206661	−.1835419	.943477	.5885621
C-Section	.0540464	−.0597158	1.135394	.8804142
Birth Interval	−.0408154	.1321761	1.135338	.7049418
Facility Delivery	.2746749	.0830562	.7614058	.9029657
Distance to Health Facility a Problem	−.1786455	.1215717	.8775627	1.140062
Employed	−.2450663	−.1205648	1.515683	1.196728
Primary	.0234105	−.0090512	1.036952	.9856519
Secondary	.2972229	−.180736	1.146584	1.003001
Higher	.0924015	−.0616649	1.527182	.7932333
Married	−.1246425	.2719016	1.13	.8624613
Living Together	.1411103	−.2760325	1.319529	.7002463
Div/Wid/Sep	−.1245533	.0329835	.6440925	1.149318
Poor	.0742811	−.0427384	1.126954	.939822
Middle	.2025143	−.1443748	1.456805	.8293347
Rich	.2487801	−.0267813	1.756897	.9556369
Richest	−.0371579	.0262596	.926871	1.056912
Urban	−.1870417	−.0771981	1.079494	1.024072
Western	.1767931	−.1432928	1.761079	.7223761
Central	.0278805	−.0476522	1.091104	.8685413
Volta	.3894944	.088634	8.038964	1.302943
Eastern	.1335153	−.0792723	1.561523	.8110256
Ashanti	.3823853	.0773616	9.837706	1.275423
Brong-Ahafo	.538495	−.0802844	21.94662	.8581629
Northern	−.5721632	.042118	.4612771	1.108165
Upper East	.3371211	.1979067	3.502459	1.801891
Upper West	−.3843526	−.0066071	.4819179	.9822969
B. Over Identification Test				
Over identification test for covariance balance				
H_0_: Covariates are Balanced				
Chi2 (31) = 22.0718				
Prob > chi 2 = 0.8807

Following [6, 45, 46] the paper estimates the propensity scores on which women in the sample are matched into and put in two groups-women with valid health insurance and women without valid health insurance. The estimation adopted a maximum of two matches (based on the psmatch option). This means that for each score, a maximum of two matches are considered. The balancing test^2^results indicate that the treatment and control groups are very similar.

In the matching, model let ED = 1 represents a woman who has a valid national health insurance and ED = 0 represent a woman with no valid national health insurance. The treatment effect of valid health insurance is represented by TREAT for the individual women written as:

$$ {Treat}_i={Y}_i(1)-{Y}_i(0) $$

In this context, *Y*_*i*_(1) represents the risk of neonatal death if the mother has a valid national health insurance and *Y*_*i*_(0) represents the risk of neonatal death if the mother did not have a valid national health insurance. In this paper, the average treatment effect on the treated (ATET)^3^ is estimated. The ATET evaluates the outcomes for those who received the treatment. In this case the, ATET estimates the risk of neonatal death for those who had a valid national health insurance. This is represented by the equation as:

$$ ATET=E\left( Treat| ED=1\right)=E\left(Y(1)| ED=1\right)-E\left(Y(0)| ED=1\right) $$

Given that the ATET directly focuses on the actual treatment participants, it evaluates precisely the gain from a program and therefore it can help determine whether or not the program or treatment was successful [47].

To check for sensitivity of results to different estimators, the paper employs other treatment effects estimators namely regression adjustment, inverse probability weights (ipw) and inverse probability weights with regression adjustment (ipwra).^4^ These three estimators model for the non-random treatment assignment in different ways. Regression adjustment accounts for the non-random assignment by modeling the outcome (neonatal deaths in this case), ipw models the treatment assignment process and not specify a model for the outcome.

The IPWRA estimator accounts for the non-randomness in the treatment assignment by modelling both the outcome and the treatment. The estimator uses the ipw weights to estimate corrected regression coefficients that are then used to perform the regression adjustment. The IPWRA estimator is characterized by the double-robust property which ensures consistent treatment effects.

All three estimators pose the question “how would the outcomes (neonatal deaths) have changed if the mothers who had valid health insurance did not have” or “how would the outcomes have changed if the mothers who did not have valid health insurance ensured that they had valid health insurance?” The difference in these two counterfactual outcomes, also called potential outcomes precisely give the actual effect of the health insurance on neonatal deaths.

## Results and discussion

The primary objectives of this study is to assess the key determinants of neonatal mortality and to determine the impact of the national health insurance on neonatal mortality. Results from the three models estimated are shown in Table 4 below. Table 5 below also shows results from the robustness checks when other estimators are employed. From Table 4, model one specifies a basic probit which does not account for the possibility of endogeneity in national health insurance uptake. Model two shows estimations from an interaction term between place of residence and the national health insurance. The last model, which is the main model of interest shows results from the propensity score matching estimation. The estimates shows that the national health insurance scheme has a significantly negative impact on the risk of neonatal deaths in Ghana. Specifically, the risk of neonatal death is reduced by about 5% for individual mothers who have valid national health insurance compared to others without a valid health insurance. This finding is consistent with those of Barros et al. [34] and Mensah et al. [6].

**Table 4:** Estimation results: Probit, Probit with Interaction and PSM

Dependent Var: Neonatal Deaths	Model1	Model 2	Model 3
	Probit	Probit with Interaction	PSM
Woman has Valid NHIS	−0.0144*	−0.0646***	
	(0.00795)	(0.0246)	
Mother’s Age	−0.000490	−0.000528	
	(0.000586)	(0.000589)	
Child is Twin	0.000431	0.000148	
	(0.0146)	(0.0147)	
Birth Order	0.00282	0.00295	
	(0.00194)	(0.00194)	
Child is Male	7.20e-05	−3.80e-06	
	(0.00492)	(0.00492)	
Number of ANC Visits	9.29e-05	0.000106	
	(0.000970)	(0.000971)	
Delivered by CS	0.00497	0.00381	
	(0.00729)	(0.00737)	
Birth Interval	−0.00933	−0.00994	
	(0.00808)	(0.00800)	
Facility Delivery	0.00961	0.00942	
	(0.00731)	(0.00727)	
Distance to Health Facility a Problem	0.0107*	0.0117**	
	(0.00552)	(0.00554)	
Employed	−0.00617	−0.00520	
	(0.00603)	(0.00605)	
	(0.0175)	(0.0177)	
Married	−0.0137	−0.0141	
	(0.00863)	(0.00864)	
Living Together	−0.0224**	−0.0227**	
	(0.0103)	(0.0103)	
Divorced/Separated/Widowed	−0.0188	−0.0189	
	(0.0141)	(0.0141)	
Urban	−0.000404	−0.0286*	
	(0.00678)	(0.0150)	
Urban*Valid NHIS		0.0323**	
		(0.0154)	
Education Controls	Yes	Yes	
Regional Controls	Yes	Yes	
Wealth Quintiles	Yes	Yes	
Valid NHIS vs No Valid NHIS			−0.051***
			(0.007)
Observations	2905	2905	2905

Standard errors in parentheses*** *p* < 0.01, ** *p* < 0.05, * *p* < 0.1

Marginal effects presented in Models 1 and 2

**Table 5:** Regressions for Robustness Checks

	(Model 1) RA		(2)		(3)	
			IPW		IPWRA	
POmeans						
Valid NHIS = 0	0.0310***	(3.14)	0.0394**	(2.04)	0.0331***	(4.39)
Valid NHIS = 1	0.0172***	(6.67)	0.0164***	(6.64)	0.0164***	(6.64)
OME0						
Mother’s Age	−0.00292	(−0.06)			−0.0199	(−0.41)
Child is Twin	2.270***	(2.89)			4.272***	(4.97)
Child is Male	−2.867*	(−1.80)			−5.079**	(−2.56)
# ANC Visits	−0.157	(−1.01)			−0.456***	(−2.68)
Delivery by CS	2.606***	(2.59)			3.997**	(2.44)
Birth Interval	−0.534	(−0.42)			−1.080	(−0.95)
Facility Delivery	−0.580	(−0.68)			−0.837	(−0.77)
Distance to Health Facility a Problem	2.464**	(2.48)			4.442***	(4.05)
Primary	−0.604	(−0.63)			0.206	(0.21)
Secondary	−0.531	(−0.59)			−0.842	(−0.80)
Higher	0.907	(0.56)			0.189	(0.07)
Poor	2.155**	(2.28)			3.491***	(2.70)
Middle	2.463*	(1.86)			2.756	(1.44)
Rich	4.465***	(2.80)			7.368***	(3.48)
Richest	2.654*	(1.72)			4.791**	(2.46)
Employed	−1.090	(−1.38)			−1.180	(−1.59)
Constant	−4.260*	(−1.83)			−4.581*	(−1.87)
OME1						
Mother’s Age	−0.00449	(−0.20)			−0.00185	(−0.08)
Twin	−3.847***	(−15.51)			−3.973***	(−15.54)
Male	0.220	(0.70)			0.259	(0.82)
# ANC Visits	0.0278	(0.41)			0.0221	(0.31)
C-Section	−0.103	(−0.21)			−0.00225	(−0.00)
Birth Interval	−0.425	(−0.87)			−0.431	(−0.88)
Facility Delivery	0.552	(1.32)			0.535	(1.30)
Distance Problem	0.431	(1.29)			0.438	(1.30)
Primary	−0.0255	(−0.05)			−0.103	(−0.22)
Secondary	0.417	(1.10)			0.393	(1.06)
Higher	−3.861***	(−8.18)			−3.982***	(−8.54)
Poor	−0.438	(−0.99)			−0.417	(−0.95)
Middle	−0.410	(−0.92)			−0.397	(−0.91)
Rich	−0.707	(−1.28)			−0.692	(−1.24)
Richest	−0.395	(−0.69)			−0.450	(−0.78)
Employed	−0.307	(−0.86)			−0.347	(−0.96)
Constant	−3.981***	(−4.31)			−4.039***	(−4.28)
TME1						
Mother’s Age			−0.0190**	(−2.38)	−0.0190**	(−2.38)
Twin			−0.219	(−1.03)	−0.219	(−1.03)
Birth Order			0.0652**	(2.44)	0.0652**	(2.44)
Male			0.00229	(0.03)	0.00229	(0.03)
# ANC Visits			0.0114	(0.73)	0.0114	(0.73)
C-Section			0.0101	(0.09)	0.0101	(0.09)
Birth Interval			0.0205	(0.16)	0.0205	(0.16)
Facility Delivery			0.0487	(0.56)	0.0487	(0.56)
Distance Problem			0.0553	(0.73)	0.0553	(0.73)
Employed			−0.242***	(−2.63)	−0.242***	(−2.63)
Primary			−0.142	(−1.39)	−0.142	(−1.39)
Secondary			0.0209	(0.21)	0.0209	(0.21)
Higher			0.308	(1.54)	0.308	(1.54)
Married			−0.0584	(−0.34)	−0.0584	(−0.34)
Living Together			0.00554	(0.03)	0.00554	(0.03)
Wid/Div/Sep			−0.355*	(−1.73)	−0.355*	(−1.73)
Poor			−0.00304	(−0.03)	−0.00304	(−0.03)
Middle			0.0667	(0.51)	0.0667	(0.51)
Rich			0.351**	(2.31)	0.351**	(2.31)
Richest			0.0376	(0.21)	0.0376	(0.21)
Urban			−0.162	(−1.63)	−0.162	(−1.63)
Western			0.942***	(5.99)	0.942***	(5.99)
Central			0.661***	(4.48)	0.661***	(4.48)
Volta			1.684***	(7.40)	1.684***	(7.40)
Eastern			0.826***	(4.95)	0.826***	(4.95)
Ashanti			1.645***	(6.71)	1.645***	(6.71)
Brong_Ahafo			2.083***	(7.60)	2.083***	(7.60)
Northern			0.0603	(0.42)	0.0603	(0.42)
U_East			1.379***	(7.61)	1.379***	(7.61)
U_West			0.188	(1.30)	0.188	(1.30)
Constant			1.202***	(3.27)	1.202***	(3.27)
Observations	2905		2905		2905	

*t* statistics in parentheses * *p* < .1, ** *p* < .05, *** *p* < .01

This finding can be explained by two possible paths- direct and indirect. Directly, the health insurance significantly reduces out of pocket payments during pregnancy until after the first post-natal check. As such any complication during pregnancy and delivery that may cause neonatal distress or deaths may be detected and treated. Also, given that infections are a major cause of neonatal mortality, early detection of infections especially within the period from delivery till the first post-natal may be treated, thus reducing the risk of death of neonates. Also, health insurance works indirectly through improved maternal health care utilization and practices which are likely to reduce neonatal deaths. As found by [5, 6, 24] the national health insurance increases antenatal care attendance, increases the probability of facility delivery and skilled delivery as swell as reduced complications during delivery. These together are likely to significantly reduce the risk of losing babies in the first month of life.

Although the probit models (together with interaction term model) do not provide impact evaluation estimates, they provide some useful information on the determinants of neonatal mortality. As expected, the probit estimations suggest a negative relationship between health insurance and the probability of neonatal deaths. Also, distance to the nearest health facility is an important determinant of neonatal mortality. Respondents who reported that distance to the nearest health facility is a problem are significantly more likely to experience neonatal deaths. The risk of death of neonates from individual mothers who complained about the distance to the health facility is approximately 1.1% higher. Most health facilities, especially in the rural areas, are located in district capitals and in relatively bigger towns. This coupled with the generally bad road network and conditions in these remote areas may make it relatively difficult to access critical health services for neonates, especially in times of emergencies, thereby increasing the risk of deaths. Especially in the rainy season when vehicles are unable to ply the roads, it makes traveling to the hospital even more precarious.

With respect to marital status and risk of neonate death, those who are married and those living-together are associated with a reduced risk of neonatal death. Individual women who are married have a 1.4% reduced risk of death while those living together have a reduced risk of 2.3% compared to single women. In order to evaluate the possible heterogeneity in effects of the national health insurance and neonatal death, an interaction between national health insurance and place of residence was taken. Results from the interaction estimation suggest that respondents who live in the urban areas and also have valid health insurance are associated with a significantly higher risk (3%) of neonatal deaths compared to others who reside in the urban areas and do not have valid cards and others who live in the rural areas. A possible explanation for this finding is that due to the increased maternal health care utilization as a result of the introduction of the health insurance scheme, there is a lot of ‘pressure’ on the hospital facilities, which renders the health care received relatively poor. This generally creates very long waiting queues which generally discourage pregnant women from patronizing essential health care services which may lead to better health outcomes of their babies. Also, the financial challenges of the health scheme has resulted in non-payment of claims by the health centers. As such, card holders are denied services. These may work together to deteriorate the neonatal outcomes in the urban centers.

Surprisingly maternal and household factors such as education and household wealth were not statistically significant predictors of risk of neonatal death. The results also indicate that there are statistically significant differences in the risk of neonatal deaths among the regions. For instance, the risk of neonatal death for respondents who reside in the Western region is approximately 3% higher than those from the greater Accra region. Similarly, Ashanti region and Brong-Ahafo regions are also associated with 3% higher risk of neonatal deaths compared to the capital region, greater Accra.

## Conclusion

The study employs both the probit and the propensity score matching technique to estimate the impact of the national health insurance on neonatal deaths. While the probit estimate provides correlates of the risk of neonatal deaths, the propensity score matching approach employed in this context empirically compares the probability of neonatal death among individuals who have active national health insurance to the probability of neonatal deaths among individulas who do not have active or valid health insurance. Our results show that neonates of mothers with valid health insurance cards are significantly less likely to die. Results from this study, therefore, suggest that the national health insurance has the potential to significantly reduce the risk of neonatal deaths. Through the national health insurance health care services in general and, neonatal health services in particular, become more affordable to the population. As a result, health officials are able to avert any health risks of neonates that are likely to result in neonatal deaths. On the contrary, however, the results also indicate that distance to health facilities increases the risk of neonatal deaths. With a valid health insurance, neonates may still be at risk of death if the distance to the nearest hospital is a problem. Also, results from the interaction between health insurance and urban residence suggest that individuals in the urban areas who have national health insurance are significantly more likely to lose their babies in the infant stages. A possible reason for this finding is that most urban health facilities are over-stretched in terms of patients served and given the recent financial challenges of the national health insurance scheme, individuals who have the health insurance are either turned away or are given relatively substandard health care services.

These, therefore, suggest that although the national health insurance scheme may offer some potential to achieving the objectives of the Ghana National New-Born Health strategy of significantly reducing neonatal deaths by the year 2018, requisite infrastructure and appropriate policy changes need to be put in place. First, there is the need to review and extend coverage of the national health insurance beyond the first postnatal care as is the case in its present state as well as other essential drugs needed for critical neonatal health care. Secondly, it may be very beneficial for the national health insurance authority to intensify its efforts in solving the financial challenges the scheme is currently facing in order to honour its financial obligations. This may provide the necessary environment for neonates to receive the optimal healthcare services required to live past the first 28 days of their lives.

## Abbreviations

ATET: Average Treatment Effect on the Treated
CHI: Community Health Insurance
FMC: Free Maternal Care
GDHS: Ghana Demographic and Health Survey
HIPC: Highly Indebted Poor Country
IPW: Inverse Probability Weights
IPWRA: Inverse Probability Weights with Regression Adjustment
MDG: Millennium Development Goals
MHO: Mutual Health Organizations
MICS: Multiple Indicator Cluster Survey
MOH: Ministry of Health
NHIA: National Health Insurance Authority
NHIS: National Health Insurance Scheme
UNICEF: United Nations International Children Emergency Fund
UN-IGME: United Nations Interagency Group Child Mortality Estimation

## Footnotes

2 Balancing Summary statistics, plots and over identification test provided in appendix Fig. 1

3 The ATET was estimated using the teffects STATA command with the psmatch option.

4 Results of these estimators are shown in the appendix in Table 3
